# Harmonic phase in polar liquids and spin ice

**DOI:** 10.1038/s41467-017-02102-1

**Published:** 2017-12-12

**Authors:** Steven T. Bramwell

**Affiliations:** London Centre for Nanotechnology and Department of Physics and Astronomy, University College London, 17-19 Gordon Street, London, WC1H 0AJ UK

## Abstract

Many liquid or liquid-like states remain stable down to temperatures well below the interaction energy scale, where mean-field theory predicts an ordering transition. In magnetism, correlated states such as spin ice and the spin liquid have been described as Coulomb phases, governed by an emergent gauge principle. In the physical chemistry of polar liquids, systems that evade mean field order have, in contrast, been described by Onsager’s theory of the reaction field. Here we observe that in the low-temperature limit, Onsager’s theory may be cast as a prototypical theory of the Coulomb phase. However at finite temperature, it describes a distinct geometrical state, characterised by harmonic functions. This state, labelled here the ‘harmonic phase’, is shown to occur experimentally in spin ice, a dipolar lattice system. It is suggested to be relevant to more general dipolar liquids.

## Abstract (web summary)

Spin ice materials can be described using idealised models of frustrated magnetism and have motivated a revisiting of the theory of interacting dipolar systems. Bramwell shows that Onsager’s theory of polar liquids describes the Coulomb phase and predicts a distinct ‘harmonic phase’ at finite temperature.

## Introduction

**Fig. 1:**
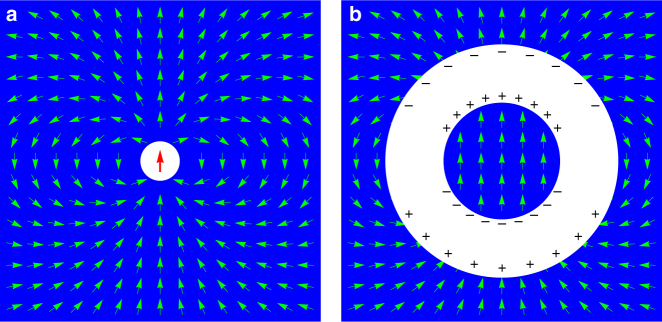
Onsager’s model of a polar liquid. **a** The model consists of a spherical cavity of molecular dimensions (white) in a continuous polarisable medium (blue). An orientable dipole (red) located at the centre of the cavity polarises the medium (green) through its dipole field. An applied field (not shown) may also be present. The nontrivial properties of the model arise from the solution of the Laplace equation in this context and the corresponding statistical mechanics^2^. **b** A blow-up of the cavity to illustrate how the central dipole may be replaced by a uniformly polarised sphere with a variable radius. Surface charges on the sphere and cavity walls are indicated

Polar liquids constitute a major class of condensed matter: well-known examples include water, acetone and nitrobenzene. Motivated by experimental observations^1^, Onsager noted that the dielectric susceptibility *χ* = *P*/$\epsilon _0$*E* of such systems (where *E* = electric field, *P* = polarisation) tends to behave as *χ* ≈ constant/*T*, rather than the steeply varying function of temperature predicted by Debye’s mean field theory^2^. Thermodynamically this implies that the energy of the system depends only weakly on the polarisation. To capture this behaviour, Onsager constructed a deceptively simple model of dipole–dipole interactions (Fig. 1a): a point dipole is located at the centre of a spherical cavity of molecular dimensions in a continuous polarisable medium. Solving the Laplace equation he found that the reaction field induced on the dipole by its own presence is always parallel to the dipole moment, so does no work in aligning it. The thermal average of the dipole moment thus experiences zero mean field and the system does not order at finite temperature. However the dipole remains correlated with the surrounding medium because of the constraint of the Laplace equation. The result is that, even for a rigid dipole (assumed in the present paper) the Curie law constant *C* of the non-interacting case is multiplied by a weakly temperature-dependent factor *γ* = (3*χ* + 3)/(2*χ* + 3). Hence the adjusted Curie law *χ* = *γC*/*T*.

In its fullest form, Onsager’s model allows for the molecule’s polarisability, as well as its dipole moment. The polarisibility *α* is introduced as the usual (Lorenz–Lorentz or Clausius–Mossotti) function of the ‘internal’ refractive index *n* and the molecular volume, (4/3)*πa*^3^. For this purpose, the hard-sphere molecular radius *a* is considered to be identical to the cavity radius *a*. The bulk susceptibility and the bulk dielectric constant (relative permittivity) $\epsilon$ = 1 + *χ* additionally depend^2^ on the molecular dipole moment *p*, as well as the calculated function *γ* (these are respectively labelled *μ* and μ^*^/*μ* by Onsager^2^).

A surprising aspect of Onsager’s theory is that, in response to applied fields, the molecular dipole moment manifests as *γ***p** rather than the expected ${\bf{p}} = \mathop {\sum}\nolimits_i q_i{\bf{r}}_i$, where *q*_*i*_, **r**_*i*_ are the charges and their positions within the molecule. Onsager called *γ***p** the ‘external moment’ of the molecule. He found that the potential energy of the molecular dipole in a field **E** is −*γ***p**⋅**E** rather than the usual −**p**⋅**E** and this translates through to thermodynamic properties once derivatives with respect to field are taken. The factor *γ* in the external moment arises from the modification of an applied (homogenous) field by the cavity.

As a simple method of relating the thermodynamic quantities *χ*, $\epsilon$ to the molecular quantities *p*, *α*, Onsager’s model has been widely and successfully applied. In this practical sense it is generally considered to be superior to Debye’s model. It was further developed, following Kirkwood^3^, into more realistic and rigorous descriptions of real liquid statistical thermodynamics. More recently, the relationship of Onsager’s, Debye’s and other approaches to the polar liquid has been clarified in the context of the hard sphere dipole fluid^4^. The reaction field concept has also been applied to magnetic systems, including spin glasses^5^ and low dimensional magnets^6^.

In the following we examine the suppression of mean field order in the Onsager picture by considering the Helmholtz–Hodge decomposition of the vector fields involved. After defining an ideal polar liquid, and developing a ‘filled cavity’ variant of the Onsager theory, correlation and scattering functions are derived. In the low temperature limit, the theory is found to describe a Coulomb phase^7,8^, while at finite temperature, it describes an unexpected state—the harmonic phase. When applied to spin ice, a magnetic system, this result is found to eliminate a discrepancy between theory and experiment. An equivalent linear response approach emphasises how the coexistence of the harmonic phase with spin ice’s magnetic ‘monopoles’ is a kind of ‘fragmentation’^9^, while the Onsager cavity is seen to account for the fundamental inhomogeneity (or discreteness) of condensed matter on the atomic scale.

## Results

### The ideal polar liquid

We define an ‘ideal polar liquid’ as a hypothetical system of permanent dipoles that obeys Onsager’s theory^2^ over the entire temperature range. Once the polarisability is suppressed, the cavity radius *a* becomes a largely superfluous parameter; it can however be retained to give some sense of the extent of a real molecule, for example through its ‘form factor’ (see below). The more general question of whether or not this model is a good starting point by which to describe real polar liquids is set aside for later discussion.

The ideal polar liquid, thus defined, exhibits a simple thermodynamic behaviour: in the high temperature limit, its susceptibility approaches that of the non-interacting system, characterised by the Curie law, *χ* = *C*/*T*, while in the low temperature limit, it approaches *χ* = (3/2)*C*/*T*, which may be shown by solving the implicit equation *χT*/*C* = *γ*(*χ*).

The crossover from *γ* = 1 at high temperature to *γ* = 3/2 at low temperature may be understood in terms of the correlations of the vector fields of the system. To analyse this, it will be useful to recall that a vector field may be Helmholtz decomposed into a divergence-free (solenoidal) part which is the curl of a vector potential and a curl-free (irrotational) part which is the gradient of a scalar potential. If there is no divergence or curl within a certain domain then the scalar or vector potentials are said to be harmonic in that domain: they obey the Laplace equation and are uniquely determined by their values at the boundaries of the domain^10^. The associated fields are also said to be harmonic. Occasionally it is useful to describe the vector field as having three components: divergence-full, rotational and harmonic. This is often referred to as a Helmholtz–Hodge decomposition^11^. An example of an explicit decomposition of this sort in a statistical mechanical system is given by Faulkner et al.^12^.

**Fig. 2:**
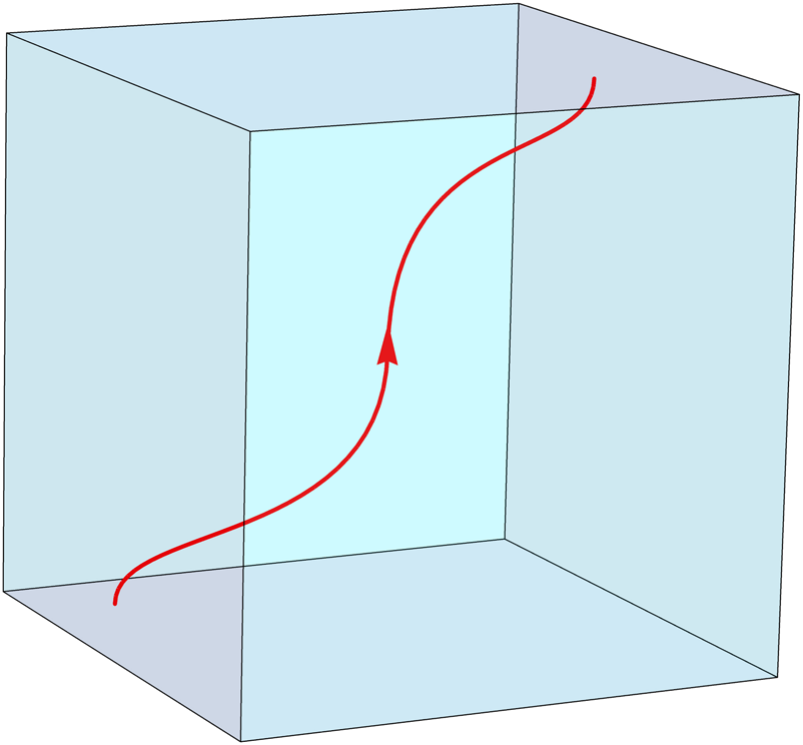
Harmonic fields. Illustration of a harmonic polarisation field line (red) in a simply connected system: the field line starts and ends on the system boundaries, with no divergence or curl within the volume of the system

The Helmholtz decomposition of the macroscopic polarisation field in electrostatics is reflected in the constitutive equation **P** = **D** − $\epsilon _0$**E**. Within a polarisable medium and in the absence of an applied field, **D** and −$\epsilon _0$**E** become equal to the solenoidal and irrotational parts of **P**, respectively. In magnetostatics the analogous constitutive equation is **M** = **B**/*μ*_0_ − **H** and hence (in the absence of applied field) **B**/*μ*_0_ and −**H** are equal to the solenoidal and irrotational parts of the magnetisation **M**, respectively. In a simply-connected domain the field lines of the harmonic polarisation or magnetisation fields (associated with either component) start and end at the system boundaries (Fig. 2).

### Filled cavity model

As a description of internal molecular structure, Onsager’s model is highly idealised: real molecules do not consist of a dipolar nucleus surrounded by empty space. There is, however, scope to alter the contents of the cavity without affecting the important properties of the model.

Following Griffiths^13^, the point dipole may be represented as a uniformly polarised sphere of radius *a*_0_ ≲ *a* that is shrunk down in size such that *a*_0_ → 0 (see Fig. 1b). The field external to the sphere is just that of the point dipole, while the limit *a*_0_ → 0 yields a delta function at the origin that represents the internal field of the dipole. The average field in the cavity has a finite contribution from the homogenous reaction field and the homogenous internal field of the sphere. Both contributions are independent of *a*_0_ and using the results of Onsager^2^ and Griffiths^13^ it is straightforward to show that the average field in the cavity (in zero applied field) is **E**_av_ = −*γ***p**/3$\epsilon \epsilon _0$*v*_0_, where *v*_0_ = (4/3)*πa*^3^ is the cavity volume. But exactly the same average field can be generated if the the cavity is filled with the same material as the medium, uniformly polarised. Theorem 1 of Griffiths^13^ may then be used to derive the corresponding dipole moment **p**′ of the filled cavity from the solution of **E**_av_ = −*p*′/3*v*_0_$\epsilon \epsilon _0$. The unsurprising result is that **p**′ = *γ***p**, the external moment.

Regarding the cavity as filled in this way preserves the important results of Onsager’s theory^2^ such as the external field, the reaction field and the average cavity field, while removing the rather complex internal field structure of the cavity plus point dipole. It gives the correct average field for any distribution of charge within the spherical cavity and is more convenient for our purposes below, where we consider Fourier transforms of the polarisation. The filled cavity, with uniform polarisation, may easily be incorporated into the Fourier transform, where it introduces a slow, form-factor like variation with wave vector *q*. However, we prefer to suppress this slow variation in order to reveal the intermolecular correlations. The filled cavity picture allows this to be done in a mathematically transparent way, by taking the limit *a* → 0. This unambiguously yields the following expressions for the electric field (**e**) and displacement field (**d**) of the central dipole:

1 $$	{\bf{e}}({\bf{r}}) = \frac{\gamma }{{4\pi \epsilon _0}}\frac{{3({\bf{p}} \cdot \widehat {\bf{r}})\widehat {\bf{r}} - {\bf{p}}}}{{r^3}} - \frac{{\gamma {\bf{p}}}}{{3\epsilon _0}}\delta ^3({\bf{r}}),\\ 	 {\bf{d}}({\bf{r}}) = \frac{\gamma }{{4\pi }}\frac{{3({\bf{p}} \cdot \widehat {\bf{r}})\widehat {\bf{r}} - {\bf{p}}}}{{r^3}} + \frac{{2\gamma {\bf{p}}}}{3}\delta ^3({\bf{r}}),$$

where the hats denote unit vectors. The left hand part of each function represents the well-known ‘outer’ field of the dipole, at finite distance from it (*r* > 0), while the delta functions represent the fields ‘within’ the dipole (*r* = 0)^13^. This special interpretation^13^ of the symbols **e**(**r**) and **d**(**r**) must be subsequently born in mind.

Equations (1) are, of course, just the usual equations for the fields of a point dipole of moment *γ***p**, the external moment. This quantity manifests whenever a response to a field is considered. In linear response theory the polarisation **P** is always understood in this context and hence, when we consider the polarisation below, it is the fields of Eq. (1) that are used to define the polarisation. Taking the limit *a* → 0 reduces the Onsager cavity to a uniformly polarised, infinitesimal sphere. There remains a charge density on the surface of this sphere, that polarises the surrounding medium.

It will be useful to obtain the Fourier transforms of Eq. (1). Let Φ(*r*, *θ*, *ϕ*) = 1/4*π*$\epsilon _0$*r*, so that the potential is *ϕ* = −*γ***p** ⋅ ∇Φ(*r*) and the dipolar field is **e** = −∇*ϕ*. The Fourier transform of Φ over the domain *r* > 0 is ${\cal F}[\Phi (r)] = 1{\mathrm{/}}\epsilon _0q^2,$ where *q* is the wave vector and it may be noted that the definition of ${\cal F}$ adopted here multiplies the dimensions by a factor of length cubed. Using the Fourier transformed gradient operator (∇ → *i***q**) and Eq. (1) it follows:

2 $${\cal F}[{\bf{e}}] = \frac{{ - \gamma {\bf{q}}({\bf{p}} \cdot {\bf{q}})}}{{\epsilon _0q^2}} - \frac{{\gamma {\bf{p}}}}{{3\epsilon _0}}, \quad {\cal F}[{\bf{d}}] = - \frac{{\gamma {\bf{q}}({\bf{p}} \cdot {\bf{q}})}}{{q^2}} + \frac{{2\gamma {\bf{p}}}}{3}.$$

Note that if we were to define the whole e-field as the gradient of the scalar potential *ϕ* then it would lack the delta function and hence factor of *γ***p**/3 above. However, this problem is avoided if the d-field is simultaneously defined as the curl of a vector potential **A** = −*γ***p** × $\epsilon _0$∇Φ, with Fourier transform ${\cal F}[{\bf{A}}] = i{\bf{q}} \times \gamma {\bf{p}}{\mathrm{/}}q^2$. Thus, ${\cal F}[{\bf{d}}] = - \gamma {\bf{q}} \times {\bf{q}} \times {\bf{p}}{\mathrm{/}}q^2$ and using the vector triple product rule, Eq. (2) (right hand term) is recovered, but with a ‘delta function’ term *γ***p** = **p**′ in place of 2*γ***p**/3. Hence the only practical consequence of defining the fields in terms of their potentials (rather than by Eq. (1)) is to shift the ‘delta function’ term entirely into the d-field, which maintains **p**′ = **d** − $\epsilon _0$**e**, as expected.

### Three-halves factor as a signature of the Coulomb phase

We now show that the factor of *γ* = 3/2, reached at low temperature in the ideal polar liquid, is a universal signature of the Coulomb phase^7,8^. This is a low-temperature state that is known from the study of spin liquids in frustrated magnets^14^.

In terms of the dipolar fields of Eq. (1), the fields **E** and **D** are^2^:

3 $$\begin{array}{l}{\bf{D}}({\bf{r}}) = {\bf{d}}({\bf{r}}),\quad {\bf{E}}({\bf{r}}) = \frac{{{\bf{e}}({\bf{r}})}}{{1 + \chi }}\quad \left( {{\mathrm{medium}}} \right),\\ {\bf{D}}({\bf{r}}) = {\bf{d}}(0),\quad {\bf{E}}({\bf{r}}) = {\bf{e}}(0)\quad \left( {{\mathrm{cavity}}} \right).\end{array}$$

Hence the polarisation is

4 $$\begin{array}{l}{\bf{P}}({\bf{r}}) = {\bf{d}}({\bf{r}}) - \frac{{\epsilon _0{\bf{e}}({\bf{r}})}}{{1 + \chi }} = \frac{{\chi \epsilon _0{\bf{e}}({\bf{r}})}}{{1 + \chi }}\quad (r > 0),\\ {\bf{P}}(0) = {\bf{d}}(0) - \epsilon _0{\bf{e}}(0) = \gamma {\bf{p}}\delta ^3(0)\quad (r = 0).\end{array}$$

The appearance of *γ***p** in place of **p** at **r** = 0 is perhaps counterintuitive, but as stressed above, it is necessary for both mathematical and physical consistency. Below it will be shown how the theory recognises that the ‘true’ dipole moment is **p** rather than *γ***p** and how all the important results of Onsager’s theory (for rigid dipoles) may be generated from corresponding linear response equations.

With the assumption of translational invariance, and after thermal averaging, the product of the components of **p** with those of **P**(**r**) of Eq. (4) determines the polarisation correlation function of the system:

5 $${\cal C}^{\alpha \beta }({\bf{r}}) = \left\langle {p^\alpha P^\beta ({\bf{r}})} \right\rangle ,$$

where *α*, *β* = *x*, *y*, *z* are Cartesian components in the laboratory frame. However, given that the correlation of the central dipole with the medium is purely mechanical at all temperatures, and that the system has spherical symmetry, the above thermal average is equal to a directional average, denoted below by a bar:

6 $${\cal C}^{\alpha \beta }({\bf{r}}) = \overline {p^\alpha P^\beta ({\bf{r}})}$$

This function may be Fourier transformed to derive the static scattering function in reciprocal space. Using Eqs. (2) and (4), and noting that *χγ*/(1 *+* *χ*) = 3*γ* − 3, the non-averaged result is

7 $${\cal F}\left[ {p^\alpha P^\beta ({\bf{r}})} \right] = \gamma p^\alpha p^\beta - (3\gamma - 3)\frac{{p^\alpha q^\beta \left( {p^iq^i} \right)}}{{\epsilon _0q^2}},$$

where ${\cal F}$ denotes a Fourier transform and summation over related indices is implicit. The angular average of this function may be resolved by noting that $\overline {p^\alpha p^\beta } = \left( {p^2{\mathrm{/}}3} \right)\delta _{\alpha \beta }$. Hence dividing by *p*^2^/3 and after some rearrangement, we finally derive the normalised static scattering function for polarisation fluctuations:

8 $$S^{\alpha \beta }({\bf{q}}) = \left( {3{\mathrm{/}}p^2} \right){\cal F}\left[ {{\cal C}^{\alpha \beta }({\bf{r}})} \right] = \gamma \left[ {\delta _{\alpha \beta } - \frac{{q_\alpha q_\beta }}{{q^2}}} \right] + (3 - 2\gamma )\left[ {\frac{{q_\alpha q_\beta }}{{q^2}}} \right].$$

The result Eq. (8) is observed to obey the total moment sum rule:

9 $$\frac{{\mathop {\sum}\limits_\alpha \mathop {\sum}\limits_{\bf{q}} {\cal F}\left[ {{\cal C}^{\alpha \alpha }({\bf{r}})} \right]}}{{\mathop {\sum}\limits_{\bf{q}} }} = p^2,$$

and hence the theory recognises that the microscopic dipole has magnitude *p*, as anticipated.

The terms in square brackets in the right hand expression of Eq. (8) are, respectively, the transverse and longitudinal projection operators. They (respectively) select components of the tensor *S*^*αβ*^(**q**) perpendicular and parallel to **q**, which in turn arise from the correlation of the central dipole with the solenoidal and irrotational components of **P**(**r**). Recalling that *γ* varies from 1 at high temperature to 3/2 at low temperature, we find that in the high temperature limit, *S*^*αβ*^(**q**) → *δ*_*αβ*_, while in the low-temperature limit *S*^*αβ*^(**q**) → (3/2)[*δ*_*αβ*_ − *q*_*α*_*q*_*β*_/*q*^2^] and the correlations become purely transverse to a given **q**. However, according to Eq. (8), the trace of the tensor *S*^*αβ*^(**q**) is independent of temperature: Tr*S*^*αβ*^(**q**) = 3. This factor 3 relates to the three Cartesian components (*x*, *y*, *z*) of **P**_**q**_, which are statistically independent for small fluctuations. Thus in both the high and low temperature limits, the polarisation is freely fluctuating with free energy $F = \epsilon _0^{ - 1}{\int} \left| {{\bf{P}}_{\bf{q}}^2} \right|{\mathrm{/}}2\chi {\mathrm d}^3q$, but at low temperature it is subject to the additional topological constraint **q** ⋅ **P**_**q**_ = 0. This means that only two of the three independent Cartesian components are thermally active; hence to maintain the trace of the tensor, its components need to be multiplied by the factor 3/2.

It also follows that at low temperature the central dipole induces a field that is purely solenoidal over the whole domain of the system (both inside and outside the cavity): **P**(**r**) = **D**(**r**) = ∇ × **A**′, with ∇ ⋅ **P** = 0. If this is the only constraint on the system then *γ* = 3/2 is the only value of *γ* consistent with free fluctuations.

These properties demonstrate that the low temperature state of the ideal polar liquid is a Coulomb phase^8^, that its polarisation is an emergent gauge field and that *γ* = *χT*/*C* = 3/2 is the universal signature of its free fluctuations. In physical terms it is hardly surprising that an ideal polar liquid excludes bound charge density (−∇ ⋅ **P**) to form a Coulomb phase, but what is notable is that this is clearly described in Onsager’s minimal model.

### Definition of the harmonic phase

Excitations out of the Coulomb phase are expected to be monopoles of bound charge density^8^ but these are not described by the Onsager model. Instead, the fields in the medium, Eq. (3), arise from potentials that satisfy the Laplace equation and hence are harmonic functions. The polarisation in the medium is therefore a harmonic field and *S*^*αβ*^(**q**) reflects this. At low temperature the central dipole **p** induces a solenoidal polarisation, as already discussed. As the temperature increases the induced polarisation becomes in part irrotational, but within the medium it remains harmonic. The solenoidal and irrotational polarisation fields give rise to distinct components in the correlation function tensor: the solenoidal component gives rise to transverse (to **q**) correlations while the irrotational component gives rise to longitudinal (to **q**) correlations. At finite temperature, the correlation function is a sum of the transverse and longitudinal components, weighted by the factors *γ*(*T*) and 3 − 2*γ*(*T*) respectively (see Eq. (8)).

**Fig. 3:**
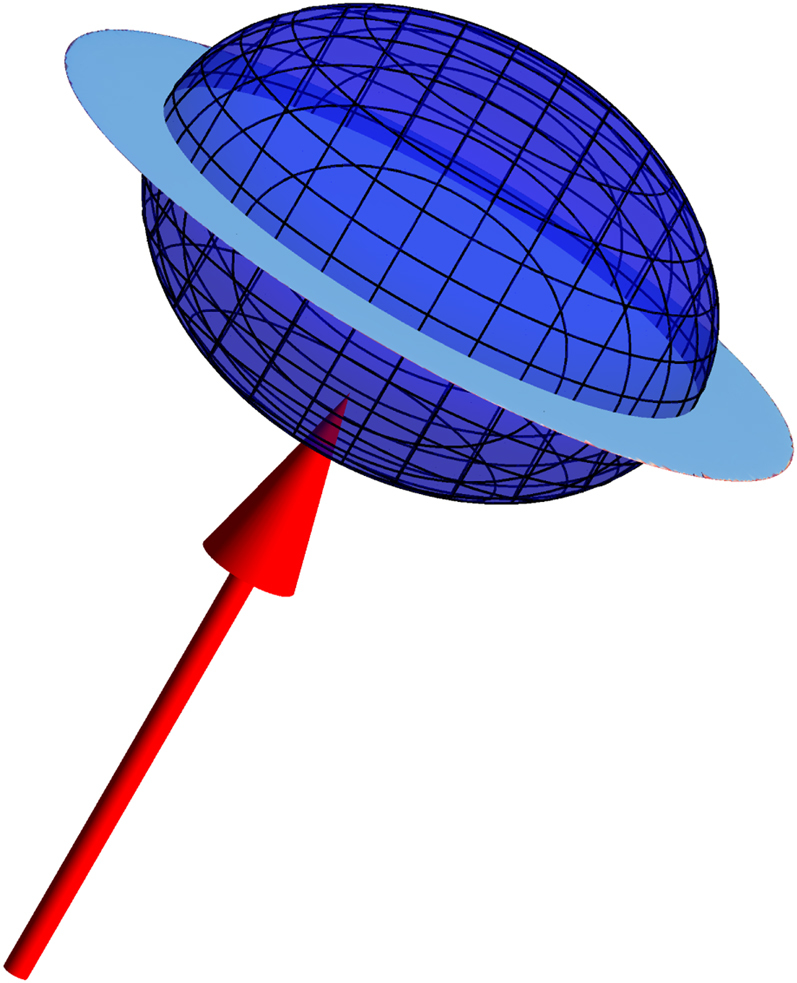
Geometrical difference between Coulomb and harmonic phases. Representation surface of the correlation function tensor *S*^*αβ*^(**q**) with respect to the wave vector **q** (red). In the Coulomb phase it is an infinitely thin disc with radius ${\sqrt{3/2}}$, but in the harmonic phase it becomes an oblate spheroid, that evolves from the disc in the low-temperature limit towards a unit sphere at high temperature (disc and example spheroid shown overlaid together)

We term such a monopole-free, curl-free state the ‘harmonic phase’ to emphasise its distinction from the Coulomb phase. The harmonic phase is geometrically different to the Coulomb phase, as illustrated in Fig. 3. In the Coulomb phase the tensor *S*^*αβ*^(**q**) has one zero and two degenerate eigenvalues and it may be represented by an infinitely thin disc of radius ${\sqrt{3/2}}$ with its axis parallel to **q**. In the harmonic phase the zero eigenvalue becomes finite and *S*^*αβ*^(**q**) is represented by an oblate spheroid, with ellipticity tending to unity at high temperature. This difference allows the correlation function to evolve continuously, without breaking any symmetry, from that of the Coulomb phase at low temperature, to that of the ideal paramagnet at high temperature.

**Fig. 4:**
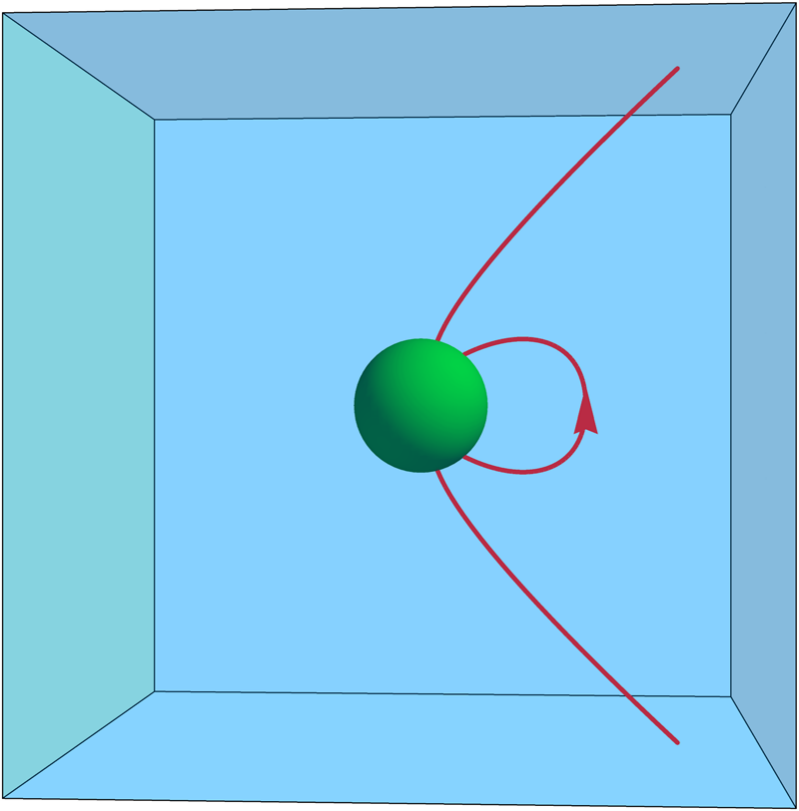
The Onsager cavity acts as an inner boundary of the system. The harmonic phase is defined by polarisation or magnetisation field lines (red) that terminate on either the inner (green) or outer system boundaries but not within the system volume (blue)

It should be noted that the designation ‘harmonic phase’ only has a strict meaning with respect to a given set of boundary conditions. In the Onsager model, the microscopic cavity acts as an ‘inner boundary’ of the medium. The polarisation field lines produced by the central dipole begin and end on both inner and outer boundaries (see Fig. 4) and are determined by the potentials there. The sources of the irrotational field components are bound charges induced by the central dipole on the surface of the Onsager cavity (Fig. 1b). As discussed further below, in an equivalent representation that eliminates the Onsager cavity, only the solenoidal fields remain harmonic in the whole domain of the medium, the irrotational fields being generated by a volume charge density within that domain.

### Linear response description of the harmonic phase

Linear response theory relates *S*^*αβ*^(**q**) to the polarisation induced by a weak applied field $\overline {\bf{E}}$. This field is notionally removed at time *t* = 0 and the dynamical correlation functions *S*^*αβ*^(**q**, *t* → 0^+^) becomes equal to $S^{\alpha \beta }({\bf{q}}) = \chi ^{\alpha \beta }({\bf{q}})T{\mathrm{/}}C = (T{\mathrm{/}}C)P_{\bf{q}}^\alpha {\mathrm{/}}\epsilon _0\overline E _{\bf{q}}^\beta$. In this representation it is convenient to treat a continuous system with periodic boundaries.

The harmonic phase may be described by the following linear response equation:

10 $${\bf{P}}({\bf{r}}) = \chi \left[ {{\int} \epsilon _0\overline {\bf{E}} \left( {{\bf{r{\prime}}}} \right)\delta \left( {{\bf{r}} - {\bf{r{\prime}}}} \right){\mathrm d}^3r{\prime} + \epsilon _0\widetilde {\bf{E}}({\bf{r}})} \right].$$

supplemented by the total moment sum rule, Eq. (9). Here ${\widetilde {\bf E}}$ = −*∇ψ* is the internally-generated E-field:

11 $$\widetilde {\bf{E}}({\bf{r}}) = - \frac{1}{{4\pi \epsilon _0}}\nabla {\int} \frac{{\rho \left( {{\bf{r{\prime}}}} \right)}}{{\left| {{\bf{r}} - {\bf{r{\prime}}}} \right|}}{\mathrm d}^3r{\prime},$$

and *ρ*(**r**) = $\epsilon _0$∇ ⋅ $\widetilde {\bf{E}}({\bf{r}})$ = −∇ ⋅ **P**(**r**) is the bound charge density. Thus (as shown explicitly below in the analogous magnetic case), solution of Eq. (10) gives Eq. (8); then enforcing Eq. (9) implies *γ* ≡ *χT*/*C* *=* (3*χ* + 3*)*/(2*χ* + 3) and hence the temperature dependence of *γ* and *χ*.

However, in this description, only the solenoidal fields remain harmonic, with field lines that connect through the periodic boundaries to form continuous loops (the equivalent in a simply connected system would be field lines like those of Fig. 2). The irrotational fields, in contrast, arise from the volume source density *ρ*(**r**), which substitutes for the surface charge in the cavity model (see Fig. 1). The Onsager cavity thus captures an essential small-scale inhomogeneity. In a truly homogenous system, the fields arising from an element of polarisation at **r** would not induce elements of volume charge density at **r**′.

A peculiar aspect of this representation may be noted: it unnecessary to specify the actual charge distribution *ρ*(**r**) but it is important that the charge density is unscreened. From this perspective, the role of the Onsager cavity is to provide an unscreened charge distribution. The effect of screening is considered further below.

### Experimental evidence of the harmonic phase in spin ice

The question arises, does the harmonic phase describe a real experimental state? This question is answered with reference to spin ice, the paradigm frustrated ferromagnet^15–20^ and a model Coulomb phase system^21–23^. The above ideas may be applied to spin ice by substituting electric properties for magnetic ones ({**P**, **D**, $\epsilon _0$ **E**} → {*μ*_0_**M**, **B**, *μ*_0_**H**}) and treating the lattice system as a liquid (see below). The largely dipolar interactions of spin ice are conventionally modelled by the near neighbour model^16^, which truncates the dipolar interaction at near neighbour, or by the the more realistic dipolar spin ice model^18,19^, which includes the full interaction. At low temperature spin ice tends to a Coulomb phase with solenoidal magnetisation;^23^ at finite temperatures, the existence of magnetic monopole excitations is well established^22,24–28^.

Before testing for the existence of a harmonic phase in spin ice there is one issue to clarify. The near neighbour model itself creates pseudo-dipolar correlations and a Coulomb phase, but in the approach to absolute zero, the magnetic susceptibility behaves^21,29–31^ as *χ* = 2*C*/*T*, rather than the universal *χ* = (3/2)*C*/*T*. According to the above argument, this can only mean that there is an another constraint on the system, in addition to ∇ ⋅ **M** = 0. To identify this constraint, we note that spin ice is formed of spin-tetrahedra at the microscopic level, in which the strongest correlations occur. Beyond this, treating spin ice as a liquid is fairly natural: it has cubic space symmetry which means that bulk linear response properties have spherical symmetry, like Onsager’s model. However it seems reasonable to place in the spherical cavity a single tetrahedron of spins, which will be treated as the dipole in Onsager’s model, its multipolar fields being neglected (a reasonable approximation^22^). This is in the spirit of Kirkwood’s refinement^3^ of Onsager’s model. Note that, although the spins are Ising-like, their energies are those of rigid, orientable dipoles, so the Onsager method still applies. As shown in the Methods, the susceptibility of a single spin ice tetrahedron (in vacuo) may be written:

12 $$\frac{{\chi T}}{C} = \frac{4}{{3 + e^{ - 2/t} - e^{4/t} + e^{ - 6/t}}}$$

where *t* = *T*/*J* and *J* is the effective exchange coupling. Plugging this in to Onsager’s implicit equation *χT*/*C* = *γ*(*χ*) and solving gives the same high and low temperature limits as the (essentially exact) calculation for near neighbour spin ice i.e. *χT*/*C* → 1, 2 respectively. The factor 2 is thus seen to arise as a product of the factor 4/3 from Eq. (12) (as *t* → 0) and the universal factor 3/2 for the Coulomb phase, as identified above: that is, it seems that the recursive calculation^30^ (that yields the factor 2) substitutes for the Onsager correction. The tetrahedron model thus illustrates how the universal (3/2) amplitude of the Coulomb phase is affected by the extra constraint of local correlations.

Having clarified this, we return to the programme of testing the validity of the harmonic phase. Our polar liquid model of spin ice infers the magnetic correlation function of the form Eq. (8) multiplied by a tetrahedron ‘form factor’ (henceforth neglected). This is nearly the same as that calculated analytically for spin ice^32,33^. The lattice nature of spin ice means that the static polarised neutron scattering function manifests as ‘pinch points’ near each Brillouin zone centre, which are indeed contained in Eq. (8). In an ideal polar liquid the absence of a reciprocal lattice means that the experimental scattering would be less striking, even though the scattering function is mathematically that of the ‘pinch point’.

There is, however, a difference in the structure factor for the harmonic phase and that predicted by the analytic calculations^32,33^ for the monopole gas. This occurs in the longitudinal term of Eq. (8). The difference may be seen by writing the two longitudinal (L) expressions side by side:

13 $$S_{{\mathrm{harmonic}}}^{\mathrm{L}}({\bf{q}}) = \frac{{\chi T{\mathrm{/}}C}}{{(1 + \chi )}},\quad S_{{\mathrm{monopole}}}^{\mathrm{L}}({\bf{q}}) = \frac{{\chi T{\mathrm{/}}C}}{{(1 + \chi ) + \xi ^2q^2}}.$$

Here *ξ* is the diffusion length for monopoles, which obeys *ξ*^2^ ∝ 1/*n* where *n* is the monopole density^33^. Except at zero *q*, the two expressions are mathematically different and represent different correlations: those of harmonic and divergence-full fields respectively. The monopole expression was derived in a low temperature approximation^33^, leaving open the possibility that the harmonic expression is relevant at more general temperatures.

**Fig. 5:**
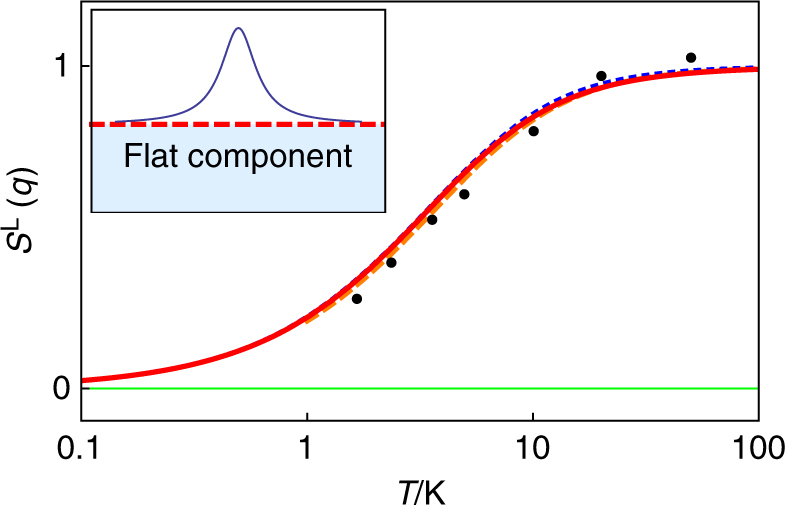
Experimental indication of the harmonic phase in spin ice. Inset: line shape of the longitudinal scattering function of Ho_2_Ti_2_O_7_, indicating the flat component beneath a Lorentzian peak. Main figure: points are experimental neutron scattering data for the ‘flat’ component of the longitudinal correlation function *S*^L^(*q*) of Ho_2_Ti_2_O_7_ measured at **Q** = (0.5, 0.5, 2) vs. temperature, as reported by Fennell et al.^23^, Fig. 4D. The three curves shown on the figure (which are almost coincident) correspond to *S*^L^(*q*) calculated for the harmonic phase according to the left hand expression of Eq. (13), using the following estimates of the susceptibility *χ*: (i: red full curve—see Methods) the single tetrahedron-Onsager calculation presented here; (ii: blue short dash curve), theoretical susceptibility for near neighbour spin ice;^30^ (iii: orange long dash curve) an analytic approximation to the experimental susceptibility^31^. The experimental neutron data, which is not measured in absolute units, has been scaled, but is consistent with the harmonic phase theory. Experimental random errors are smaller than the points, but systematic errors may be of order 10%—without a much deeper analysis of the experimental systematic errors, it is impossible to assess the significance of the observed small discrepancies between theory and experiment. What is certain, however, is that the harmonic phase theory (the curves) capture the experimental scattering much more accurately than the low-temperature monopole calculation^33^ (lower green line), which predicts zero intensity

To test for the harmonic phase in spin ice, we compare Eq. (13) with *S*^L^ measured by polarised neutron scattering^23^. A small Lorentzian peak has been interpreted as the monopole term (Eq. (13), right), but this sits on top of a ‘flat’ and temperature-dependent component that was noted to correlate with the thermal excitation of monopoles^23^. We find this flat component to be consistent with the prediction for the harmonic phase (Eq. (13), left) while the low-temperature monopole calculation (Eq. (13), right) predicts no flat component. Figure 5 illustrates the consistency of experiment and theory that confirms the harmonic phase. It seems reasonable to identify it with the temperature-dependent monopole vacuum, for which the Coulomb phase is its low temperature limit (see Fig. 3). The flat component indeed correlates with thermal excitation of monopole states^23^ but indicates a much more highly correlated state than previously envisaged (see Fig. 3).

### Correction for magnetic monopoles

The linear response description of the harmonic phase developed above may be refined to include the effect of thermally-generated bound charge. While this could apply to any dipolar system, it is particularly simple to formulate the correction for spin ice, because the bound charge in spin ice is discretised into a gas of effective magnetic monopoles^22^.

Adapting Eq. (10) and its associated notation to the magnetic case (such that $\widetilde {\bf{H}}$ = −∇*ψ*, *ρ* = −∇ ⋅ **M**) we first write the Poisson equation for the internal field potential,

14 $$ - \nabla \cdot \nabla \psi ({\bf{r}}) = \rho ({\bf{r}}).$$

and then introduce thermally generated monopoles to screen the fixed charge density. The internal field potential contributes a free energy *W*_*i*_ = *μ*_0_*Q*_*i*_*ψ* to each magnetic monopole of charge *Q*_*i*_ = ±*Q* or ±2*Q* (here *Q* is the monopole charge and single- and double charge monopoles are allowed^22^). Hence the problem to be solved reduces to solving the Poisson-Boltzmann equation with a fixed source density:

15 $$ - \nabla \cdot \nabla \psi = \rho + \mathop {\sum}\limits_i Q_in_ie^{ - \mu _0Q_i\psi /kT}$$

where *n*_*i*_(*T*) is the thermally averaged monopole density. In the Debye-Hückel (linear) approximation this becomes

16 $$ - \nabla \cdot \nabla \psi = \rho - \kappa ^2\psi ,$$

where overall charge neutrality has been asserted and $\kappa (T) = \sqrt {\mathop {\sum}\nolimits_i \mu _0Q_i^2n_i(T){\mathrm{/}}kT}$ is the reciprocal Debye length.

Operating through with ∇ and using the definitions of *ψ* and *ρ*, we find

17 $$\nabla \nabla \cdot \widetilde {\bf{H}} = - \nabla \nabla \cdot {\bf{M}} + \kappa ^2\widetilde {\bf{H}},$$

or in terms of Fourier components:

18 $$ - q^\alpha q^\beta \tilde H_{\bf{q}}^\alpha = q^\alpha q^\beta M_{\bf{q}}^\alpha + \kappa ^2\tilde H_{\bf{q}}^\beta .$$

Finally, using the Fourier transform of the magnetic version of Eq. (10), the above equation may be solved for the wave vector dependent susceptibility and hence the static scattering function:

19 $$S^{\alpha \beta }({\bf{q}}) = \frac{{\gamma (\kappa ^2 + \delta _{\alpha \beta }q_\alpha q_\beta )}}{{\kappa ^2\delta _{\alpha \beta } + q_\alpha q_\beta (1 + \chi )}}.$$

The longitudinal scattering function is then found to be a modified form of Eq. (13) (left):

20 $$S^{\mathrm{L}}({\bf{q}}) = \frac{{\chi T{\mathrm{/}}C}}{{1 + \chi \epsilon _q^{ - 1}}},$$

where $\epsilon$_*q*_ = 1 + *κ*^2^/*q*^2^ is the Debye–Hückel dielectric constant. In the absence of monopoles $\epsilon _q^{ - 1} \to 1$ and the harmonic phase result, Eq. (13) is recovered.

**Fig. 6:**
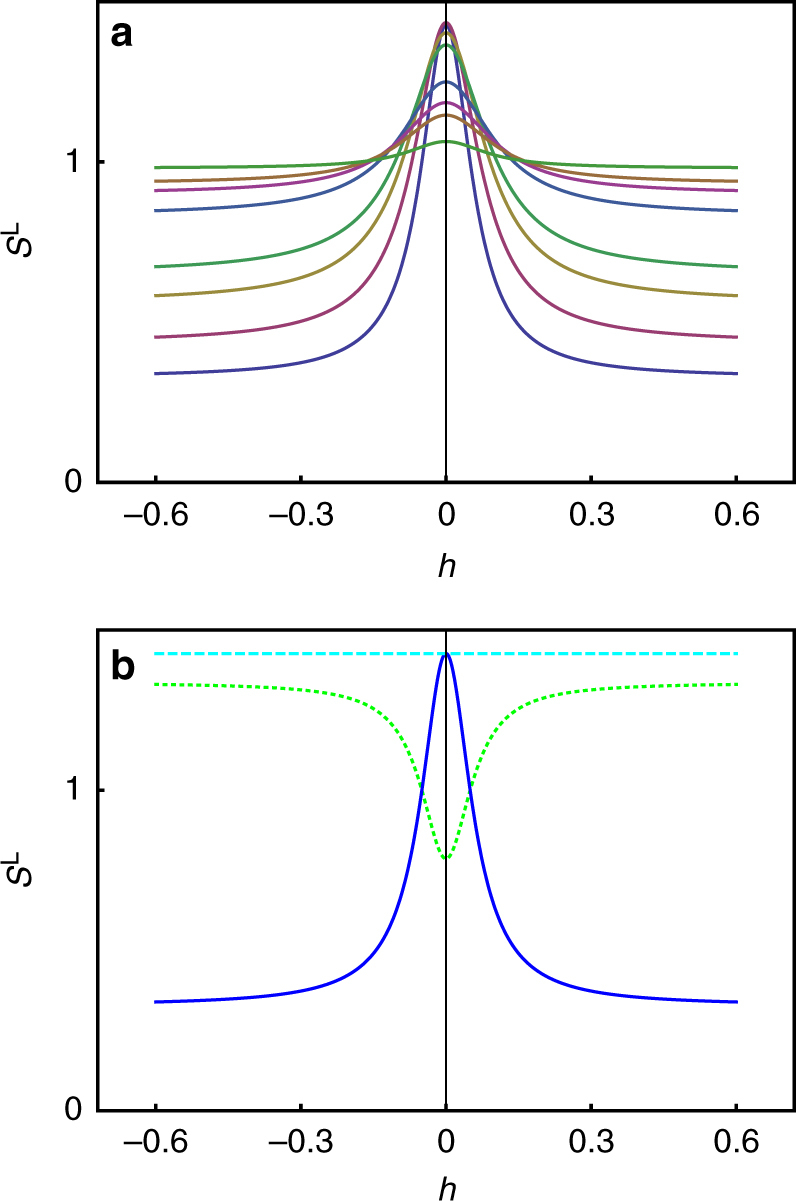
Harmonic phase corrected for monopole screening. **a** Predicted longitudinal correlation function in spin ice (Eq. (20)) along [*h*, *h*, 2] at temperatures of (top to bottom on right hand side) 50, 20, 10, 5, 3.75, 2.5 and 1.7 K. The calculation requires an input of the Debye length and susceptibility as a function of temperature. The Debye length was calculated iteratively by Debye–Hückel theory for single and double charge magnetic monopoles^49^ with excess chemical potentials appropriate to the spin ice Ho_2_Ti_2_O_7_ (*ν*_single_ = 5.7 K, *ν*_double_ = 4*ν*_single_;^22^ the bare Debye length was augmented by the distance of closest monopole approach as the standard extension of Debye–Hückel theory). The susceptibility used was an analytic approximation to the experimental data of Ho_2_Ti_2_O_7_^31^, as in Fig. 5. This plot may be compared with that of Fennell et al.^23^, Fig. 2D. The scattering separates into a flat component (the harmonic phase) and a central Lorentzian component arising from magnetic monopoles. **b** Corresponding longitudinal (blue full line) vs. transverse (cyan long dash) scattering functions from Eq. (19) at 1.7 K. The green short dash curve represents the transverse scattering function corrected to obey the total moment sum rule. This may be compared to the result of Fennell et al.^23^, Fig. 2B, C

A plot of the longitudinal scattering function, Eq. (20), as a function of temperature and wavevector is given in Fig. 6a. This looks very like the corresponding experimental data of Fennell et al.^23^, Fig. 2D. Indeed, with the identification *κ* = 1/*ξ*, Eq. (20) can easily be separated into a sum of the harmonic phase term (Eq. (13) left) and the Lorentzian monopole term (Eq. (13) right), but with the monopole term multiplied by *χ*/(1 + *χ*). In the low temperature limit, the result of monopole theory^33^ is recovered, with corrections of order 1/*χ*. A close comparison of experiment and theory is not attempted here as detailed systematic experimental corrections would need to be considered which take us far from the main point of this paper. We may conclude, however, that Eq. (20) captures the basic form of the experimental data in a way that existent monopole calculations do not, and that both experiment and theory justify the harmonic phase as a distinct component of the correlation function.

In the derivation of Eq. (19) we have essentially altered the longitudinal fields, without correcting the transverse fields to obey the total moment sum rule. Hence in contrast to Eq. (8), Eq. (19) does not obey the total moment sum rule. The transverse correlation function *S*^T^(*q*) of Eq. (19) remains the same as that of the harmonic phase, Eq. (8), as illustrated in Fig. 6b (cyan long dash line). The total moment sum rule may be restored in an ad-hoc way by requiring that 2*S*^T^(**q**) + *S*^L^(**q**) = 3, which yields the green short dash curve of Fig. 6b. This introduces a ‘dip’ in the transverse scattering function as *q* → 0, as observed in experiment^23^. Based on this, we tentatively propose that the experimental ‘dip’ is largely a consequence of the sum-rule requirement.

## Discussion

Our findings for spin ice are consistent with previous work on th`e approximation to dipolar spin ice from which monopoles are derived—the ‘dumbbell model’^22^—as interpreted by Brooks-Bartlett et al.^9^ There the magnetisation is Helmholtz-decomposed into the gradient of a scalar potential plus the curl of a vector potential, which are shown to be partly independent (‘magnetic moment fragmentation’). The present results reflect the equivalent Helmholtz-Hodge decomposition which additionally separates the harmonic field as a separate entity. The harmonic phase represents this component—which, in terms of relative weight, is a significant component of the correlation function at all temperatures. A similar Helmholtz–Hodge decomposition of the electric fields has recently been formulated for the two-dimensional Coulomb gas at the Berezinskii–Kosterlitz–Thouless transition^12^.

The present results for the magnetic susceptibility—the ‘collective Curie law’—are consistent with those of Jaubert and colleagues^30^, which associate the law with ‘topological sector fluctuations’ of the harmonic field component in the Coulomb phase. In microscopic terms the law arises from the reversal of dipole strings that traverse the entire sample^30^ (which in practice is facilitated by a small density of monopoles). The universal (3/2) amplitude found here arises when such harmonic fluctuations are the only source of susceptibility. Quantum fluctuations, and the formation of closed loops in the bulk of the medium^34^ would modify the susceptibility amplitude and cause deviations from the collective Curie law. It is interesting to note that the modification of the Curie law by dipole loop formation has recently been discussed in the context of the hard sphere magnetic polar fluid^35^.

The main testable predictions of the present work are the scattering functions of Eqs. (8), (19) and (20). The experimental signatures of the harmonic phase are the ‘flat’ longitudinal and transverse scattering functions of Eq. (8), along with their characteristic temperature dependences. In experiment, the flatness of the correlation functions might be mistaken for an absence of correlation but in fact the harmonic phase is highly correlated, as reflected in the nontrivial property of the correlation function tensor (Fig. 3).

The scattering function of Eq. (20), Fig. 6, describes a further component of the longitudinal correlation function arising from thermally generated bound charge. Even though this has been derived for the particular case of spin ice, it should be expected to apply more generally, differences between systems being represented by differing behaviour of the reciprocal Debye length, *κ*. Eq. (20) shows that as the harmonic fields propagate into the medium they are increasingly screened by thermally generated bound charge. This may be appreciated by writing the internal field in the form:

21 $${\widetilde {\bf{H}}({\bf{r}}) = - \frac{{\mu _0}}{{4\pi }}\nabla {\int} \frac{{\rho \left( {{\bf{r{\prime}}}} \right)}}{{\left| {{\bf{r}} - {\bf{r{\prime}}}} \right|\epsilon \left( {{\bf{r}} - {\bf{r{\prime}}}} \right)}}{\mathrm d}^3r{\prime} = - \frac{{\mu _0}}{{4\pi }}\nabla {\int} \frac{{\rho \left( {{\bf{r{\prime}}}} \right)}}{{\left| {{\bf{r}} - {\bf{r{\prime}}}} \right|}} e^{ - \kappa \left| {{\bf{r}} - {\bf{r{\prime}}}} \right|}{\mathrm d}^3r{\prime},}$$

where $\epsilon$ is the dielectric function in direct space (the Fourier transform of $\epsilon$_*q*_). At large distances, or *q* → 0, the effects of the small-scale inhomogeneity, or Onsager cavity, are completely screened away and the longitudinal wavevector dependent susceptibility (*χ*(**q**) = *CS*^L^(**q**)/*T*) becomes equal to the bulk susceptibility (modulo demagnetising or depolarisation effects). However the cavity remains crucial for determining the temperature-dependence of the susceptibility, as discussed above.

It would be interesting to test real polar liquids or numerical simulations thereof, to assess the relevance to those systems of the correlation functions calculated here. Of course real polar liquids^36^ are much more complex than implied by our ‘ideal polar liquid’ or even the more general Onsager model^2^, that accounts for molecular polarisability. Indeed, the Onsager model is recognised to generally ‘overcorrect’ mean field theory^37^ and a soft sphere dipolar fluid may exhibit orientational order even in the absence of crystallisation^38,39^. Nevertheless, Onsager’s model remains an important point of reference in the theory of polar liquids^40,41^. Our analysis of spin ice certainly implies that the harmonic phase is relevant to water ice, and hence, quite possibly, to water itself. We can speculate that local geometric frustration, as occurs in spin ice and water ice will generally aid the formation of the harmonic phase. Hence one could also consider its formation in some ice-rule ferroelectrics^42^, pseudo-dipolar systems like antiferromagnetic spin liquids^43^ and ‘artificial spin ice’ micro-magnetic arrays (where field-theory^44^ and reciprocal space correlations^45,46^ are of topical interest). Where such systems form Coulomb phases at low temperature, the nature of the phase transition out of the Coulomb phase to the ultimate ordered state becomes of particular interest^47,48^. By connecting Coulomb phase theory to polar liquid theory, the concept of the harmonic phase potentially broadens the class of condensed matter to which such studies are relevant.

## Methods

### Derivation of the mean square moment of a single spin tetrahedron

In the following, the magnetic moment per site is set to unity (*μ* = 1). The tetrahedron has three classes of spin configuration depending on how many spins point ‘in’ or ‘out’^20^. There are six 2:2 configurations, eight 3:1 configurations and two 4:0 configurations. If we consider the spin projection *μ*_*z*_ on *z* = [100], then a 2:2 configuration has squared dipole moment $\left( {{\sum} \mu } \right)^2{\mathrm{/}}4 = (1{\mathrm{/}}4)\left( {4{\mathrm{/}}\sqrt 3 } \right)^2 = 4{\mathrm{/}}3$ per spin, a 3:1 configuration has (1/4)2^2^ = 1 per spin and a 4:0 configuration has zero moment. Take the zero of energy at the level of the 3:1 configurations in which case 2:2 has energy −2*J* and 4:0 has energy 6*J*. The average squared tetrahedron moment is

22 $$\left\langle {\mu _z^2} \right\rangle = \chi T{\mathrm{/}}C = \frac{{(4{\mathrm{/}}3)6e^{2\beta J} + 8}}{{6e^{2\beta J} + 8 + 2e^{ - 6\beta J}}} = 4 \times \frac{{1 + e^{2\beta J}}}{{4 + 3e^{2\beta J} + e^{ - 6\beta J}}},$$

which rearranges to Eq. (12).

### Calculation of the tetrahedron susceptibility by the Onsager method

The curves in Fig. 5 are calculated from the Eq. (13) using three different estimates of *χ*. The red curve is found by first solving the implicit equation:

23 $$\frac{{\chi T}}{C} = \frac{{(3\chi + 3)}}{{(2\chi + 3)}} \times \frac{4}{{\left( {3 + e^{ - \frac{{2J}}{T}} - e^{ - \frac{{4J}}{T}} + e^{ - \frac{{6J}}{T}}} \right)}}$$

The solution is *χ* = *A* + *B* where

24 $$A = \frac{{12Ce^{6x} + 3Te^{2x} - 3Te^{4x} - 9Te^{6x} - 3T}}{{4T\left( { - e^{2x} + e^{4x} + 3e^{6x} + 1} \right)}},$$

25 $$B = \frac{{\sqrt {9\left( {T\left( { - e^{2x} + e^{4x} + 3e^{6x} + 1} \right) - 4Ce^{6x}} \right)^2 + 96CTe^{6x}\left( { - e^{2x} + e^{4x} + 3e^{6x} + 1} \right)} }}{{4T\left( { - e^{2x} + e^{4x} + 3e^{6x} + 1} \right)}},$$

and *x* = *J*/*T*. The susceptibility *χ* is then calculated using *C* = 4 K, *J* = 1.8 K for Ho_2_Ti_2_O_7_^20,31^.

### Data availability

The data sets generated and analysed in this study are available from the corresponding author on reasonable request.
