# Author Correction: Harmonic phase in polar liquids and spin ice

**DOI:** 10.1038/s41467-018-03815-7

**Published:** 2018-04-16

**Authors:** Steven T. Bramwell

**Affiliations:** London Centre for Nanotechnology and Department of Physics and Astronomy, University College London, 17-19 Gordon Street, London, WC1H 0AJ UK

Correction to: *Nature Communications* 10.1038/s41467-017-02102-1, published online 12 December 2017

The original version of this Article contained an error in the third sentence of the penultimate paragraph of the ‘Definition of the harmonic phase’ section of the Results, which incorrectly read ‘In the Coulomb phase the tensor S^*αβ*^(**q**) has one zero and two degenerate eigenvalues and it may be represented by an infinitely thin disc of radius 3/2 with its axis parallel to **q**.’ The correct version states ‘$\sqrt {3{\mathrm{/}}2}$’ in place of ‘3/2.’

Similarly, the second sentence in the legend of Fig. 3 originally incorrectly read ‘In the Coulomb phase it is an infinitely thin disc with radius 3/2, but in the harmonic phase it becomes an oblate spheroid, that evolves from the disc in the low-temperature limit towards a unit sphere at high temperature (disc and example spheroid shown overlaid together).’ The correct version states ‘$\sqrt {3{\mathrm{/}}2}$’ instead of ‘3/2’.

This has been corrected in both the PDF and HTML versions of the Article.

